# Small size apolipoprotein(a) isoforms enhance inflammatory and proteolytic potential of collagen-primed monocytes

**DOI:** 10.1186/s12944-019-1106-4

**Published:** 2019-08-31

**Authors:** Nadia Sabbah, Stéphane Jaisson, Roselyne Garnotel, Eduardo Anglés-Cano, Philippe Gillery

**Affiliations:** 1 University of Reims- Champagne-Ardenne, CNRS, MEDyC UMR 7369, Reims, France; 2 Laboratory of Biochemisry-Pharmacology-Toxicology, University Hospital of Reims, Maison Blanche Hospital, Reims, France; 3 Inserm UMR_S1140 “Innovative Therapies in Haemostasis”Faculté de Pharmacie de Paris, Paris, France; 4 Endocrinology and Metabolic Diseases Department, Cayenne hospital, Cayenne, French Guiana; 5 Clinical Investigation Center Antilles French Guiana (INSERM CIC 1424), Cayenne, French Guiana

**Keywords:** Apolipoprotein(a), Monocytes, Collagen, Atherosclerosis, Lipoprotein(a)

## Abstract

**Background:**

Atherosclerosis is an inflammatory process involving activation of monocytes recruited by various chemoattractant factors, among which lipoprotein(a) and its specific apolipoprotein apo(a). Lp(a) contains a specific apolipoprotein apo(a) which size is determined by a variable number of repeats of a specific structural domain, the kringle IV type 2 (IV-2). Lp(a) plasma concentration and apo(a) size is inversely correlated, and smaller apo(a) are major risk factors for coronary heart disease.

**Design and methods:**

The aim of this study was to evaluate the effect of recombinant apo(a) isoforms (containing 10, 18 or 34 kringles) on monocytes interacting with type I collagen.

**Results:**

Apo(a) isoforms stimulated reactive oxygen species (ROS) and matrix metalloproteinase-9 (MMP-9) production by monocytes, and not modified monocytes adhesion on type I collagen. This effect was specific of apo(a) since no effect was observed in the presence of plasminogen and was inversely related to apo(a) size. The lysine analogue 6-aminohexanoic acid which blocks the lysine binding sites (LBS), and carboxypeptidase B (CpB) which cleaves carboxy-terminal lysine residues, abolished apo(a)-induced ROS and MMP-9 production, highlighting an effect mediated by apo(a) lysing-binding sites.

**Conclusions:**

These results indicate that activation of collagen-primed monocytes stimulated with apo(a) is a Kringle number-dependent effect and reinforce the hypothesis of a role for small size apo(a) isoforms in atherothrombosis.

## Introduction

A characteristic feature in the development of atherosclerotic lesions is the accumulation of foam cells, i.e. monocyte-derived macrophages that store cholesteryl esters, resulting from low-density lipoprotein (LDL) uptake [1]. An LDL-like lipoparticle, lipoprotein(a) (Lp(a)), may act as an active factor for monocyte recruitment trough interaction with Mac-1 integrin through interaction with Mac-1 integrin [2]. Lp(a) contains a specific apolipoprotein apo(a), which size is genetically determined by a variable number of repetitions of specific structural domains named kringles (K). Apo(a) contains a kringle V (KV) and 10 subtypes of KIV, including KIV2 (KIV-2) which could be repeated (up to about 40 copies). This size polymorphism of apo(a) in serum results in the identification of the LPA gene as the major determinant for Lp(a) concentrations and represents a rare type of copy number variation [3]. It has been demonstrated that apo(a) size and Lp(a) plasma concentrations are inversely correlated. But this pattern is complex due to a considerable variation between individuals in Lp(a) levels for a given apo(a) size [4]. Elevated Lp(a) plasma concentration has been described as an independent risk factor for the development of atherosclerotic disorders, such as coronary heart disease [5–7] and recent studies showed an independent association with an increased risk of abdominal aortic aneurysm [8] and of ischemic stroke, especially relevant for young patients [9]. Clinical evidences suggest that apo(a) may be involved in pathogenic events, since an inverse correlation has been established between apo(a) size and the increased occurrence of coronary artery lesions [4, 10, 11]. Besides it has been shown that low molecular weight apo (a) isoforms were associated with a high risk of major adverse cardiovascular events within 15 years after coronary artery bypass [12]. Recently Langsted and al. described in a Danish population a high risk of mortality associated with high concentrations of lipoprotein(a), related to low KIV-2 number of repeats rather than to high cholesterol content [13]. Even though it is difficult to extrapolate these results to the general population because the study was made only with white individuals of Danish population, similar results were obtained in the Interheart study, showing that higher Lp(a) concentrations were associated with an increased risk of myocardial infarction, in particular in South Asian and Latin American populations [14].

The ability of high Lp(a) value to predict ischemic myocardial infarction risk in the general population is not modified by inflammation or food intake [15]*.* However, the molecular mechanisms supporting its involvement in the promotion of atherogenic events have not yet been fully identified. Apo(a) could bind oxidized phospholipids and has greater propensity to localize in blood vessel walls to interact with Lysine Binding Sites (LBS) and fibrin, responsible for a thrombogenic effect by inhibition of plasmin activity [16, 17].

Most of acute cardiovascular events result from disruption of atherosclerotic plaques that release thrombogenic components. Since ruptured fibrous caps are often heavily infiltrated by foam cells, it has been suggested that monocytes/macrophages played a pivotal role in plaque rupture because of their involvement in extracellular matrix (ECM) remodelling [1]. Accordingly, it has been hypothesized that interactions between inflammatory cells, ECM components and lipoproteins triggered a cascade of complex molecular events, leading to arterial wall remodelling and atherosclerosis progression.

Our group has previously shown that type I collagen, the major ECM component, modulated monocyte functions through interaction with α_x_β_2_ integrin [18]. Since apo(a) has been shown to behave as a potent chemo attractant for monocytes [2], the present study was designed to determine its potential effect on monocytes activated by type I collagen and the role of kringle IV repeats in this process.

## Materials and methods

### Preparation of type I collagen

Type I collagen was extracted from Sprague Dawley rat tail tendons (Depré, Saint-Doulchard, France) as previously described [18, 19]. Collagen preparations were verified endotoxin-free (< 0.05 endotoxin unit/ml, Limulus amebocyte lysate kinetic-QCL kit, Cambrex BioSciences, Emerainville, France).

### Production of recombinant apo(a)

The plasmids pCMV-A10, −A18, −A34 were transfected by electroporation into the adenovirus-transformed human embryonic kidney cell line 293 and recombinant apo(a) isoforms present in culture medium supplemented with proteinase inhibitors (20 U/ml aprotinin, 0.5 mM aminoethyl-benzene-sulfonylfluoride, 2 mM EDTA, and 0.01% (w/v) NaN_3_) were purified as described elsewhere [20]. The absence of proteolysis was controlled by amino terminal analysis of r-apo [a] purified from the culture medium by affinity chromatography on an immobilized monoclonal antibody directed against apo(a). Purified apo(a) isoforms were dialyzed against 0.15 M phosphate buffer (pH 7.4) and stored at − 80 °C until use. Recombinant apo(a) isoforms containing 10, 18 or 34 K (i.e.10, 18 and 34 kringle repeats, respectively) were used in this study. Before use, the absence of endotoxin was checked in each apo(a) solution, as described above.

### Monocyte isolation and culture

Whole blood was obtained from healthy volunteers after informed consent, Laboratory of Hematology, University Hospital, Reims (blood from healthy blood donors). All the donors were aware that their blood could be used for transfusion and for potential analyses and research (they had the right to refuse as provided by French Law) and peripheral monocytes were isolated by counterflow centrifugal elutriation [18]. Each experiment was performed in quadruplicate and repeated three times with homogeneous monocyte preparations obtained from three different donors. The purity of cell preparations, assessed by evaluation of the number of CD14 positive cells, was higher than 95%, and cell viability assessed by trypan blue exclusion test was higher than 98%. All experiments were performed using Ultra-Culture medium (Cambrex BioSciences, Emerainville, France). 24- or 96-well culture plates were used either untreated or coated with type I collagen, fibronectin or bovine serum albumin (controls) and washed three times with Dulbecco’s solution (137 mM NaCl, 2.7 mM KCl, 30 mM HEPES, 10 mM Glucose, 1.3 mM CaCl_2_, 1 mM MgCl_2_, pH 7.4) before use.

### Monocyte stimulation by apo(a)

The effect of apo(a) on monocytes was evaluated by incubating cells with apo(a) isoforms of different sizes at variable concentrations (20 to 200 nM). To study the mechanism of interaction between apo(a) and collagen-primed monocytes, experiments were performed either by adding (simultaneously) 100 mM 6-aminohexanoic acid (AHA) or by replacing apo(a) with 100 nM plasminogen (Calbiochem-EMD Chemicals, Gibbstown, NJ, USA) and 10 μM amiloride (Sigma, St Louis, MO, USA). In another set of experiments, monocytes were pretreated with 200 μg/ml carboxypeptidase B (CpB, Sigma) in Dulbecco’s solution for 1 hour at 37 °C under gentle shaking.

### Reactive oxygen species (ROS) production

ROS production was evaluated by incubating 1.5 × 10^5^ monocytes in Dulbecco’s solution containing 16.7 μM nitroblue tetrazolium (NBT) for 2 h at 37 °C in 96-well coated plates. NBT reduction was assessed by absorbance measurement at 560 nm [19].

### MMP-9 secretion

Total MMP-9 (pro and active forms) secreted in culture medium by 10^6^ cells for 48 h at 37 °C was measured by ELISA (Quantikine Human (total) MMP-9, R&D Systems Europe, Lille, France).

### Statistical calculations

All results have been expressed as ratios to the control series, in order to minimize the inter-donor variability of monocyte preparations. They were obtained from four independent experiments performed each in triplicate, using different preparations of monocytes. Differences of means were considered significant at *p* ≤ 0.05 using Student’s t-test.

## Results

### Apo(a) enhances ROS production by collagen-primed monocytes

**Fig. 1:**
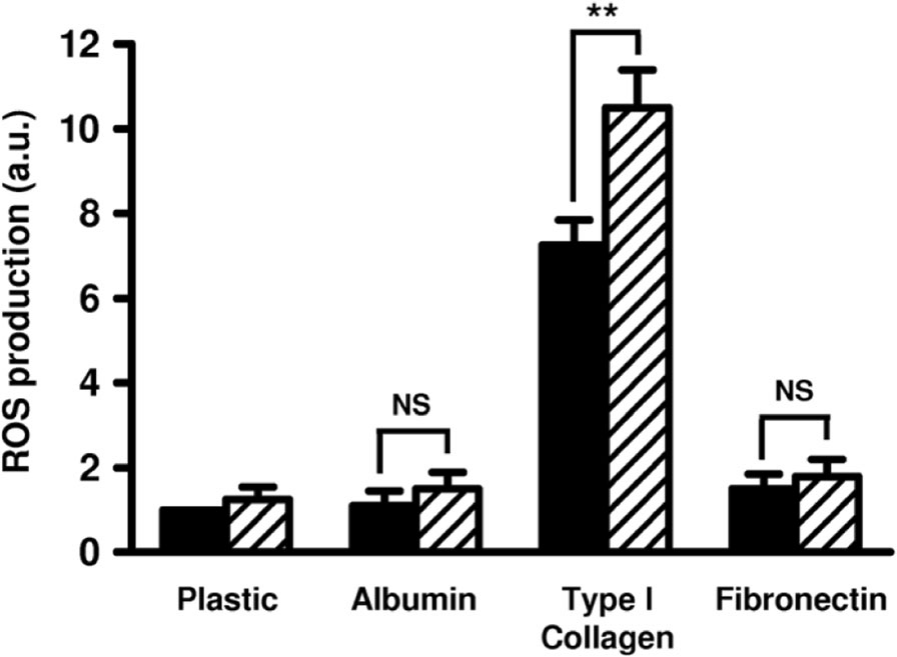
Effect of apo(a) on ROS production by monocytes seeded on different substrata. Monocytes were incubated with (hatched bars) or without (closed bars) 200 nM 18 K apo(a) for 2 h at 37 °C, on plates coated or not with albumin, type I collagen or fibronectin, and ROS production was measured by NBT reduction test. Results (means ± standard deviations) are expressed as ratios to the NBT reduction rate of monocytes incubated on plastic without apo(a). a.u.: arbitrary units, NS: non-significant, **: *p* < 0.01

**Fig. 2:**
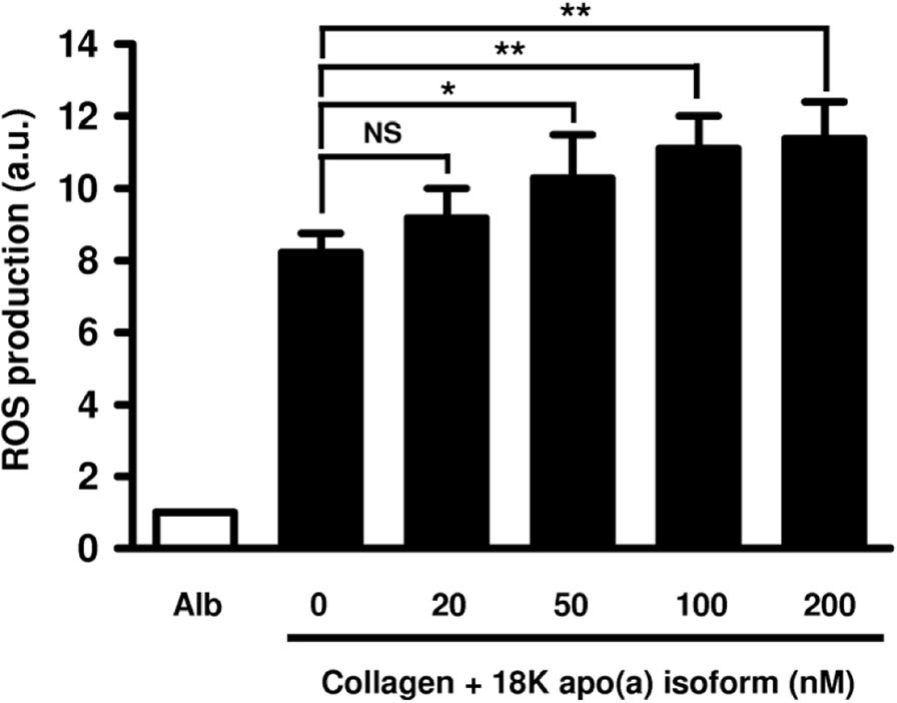
Dose-dependent effect of apo(a) on ROS production by collagen-primed monocytes. Monocytes were incubated for 2 h at 37 °C in plates coated with albumin (Alb) or type I collagen with increasing concentrations (0 to 200 nM) 18 K apo(a) isoform, and ROS production was measured by NBT reduction test. Results (means ± standard deviations) are expressed as ratios to the NBT reduction rate of monocytes incubated on albumin-coated plates. a.u.: arbitrary units, NS: non-significant, *: *p* < 0.05, **: *p* < 0.01

Type I collagen as fibronectin and albumin were efficient for monocyte adhesion (data not shown). However, only type I collagen induced ROS production by monocytes (Fig. 1). Apo(a) did not modify monocyte adhesion on any of the substrata (data not shown) nor induced ROS production by monocytes cultured on fibronectin, albumin or plastic (used as negative control of activation) (Fig. 1). Addition of apo(a) (18 K isoform) significantly enhanced ROS production induced by type I collagen in a dose-dependent manner, ranging from + 11% for 20 nM (*p* = 0.12, non-significant) to + 39% for 200 nM (*p* < 0.01) (Fig. 2). As results obtained with 100 nM or 200 nM apo(a) were not significantly different, the concentration of 100 nM was used in further experiments.

### Apo(a) enhancement of ROS and MMP-9 production by collagen-primed monocytes is inversely related to the number of Kringle IV-2 repeats

**Fig. 3:**
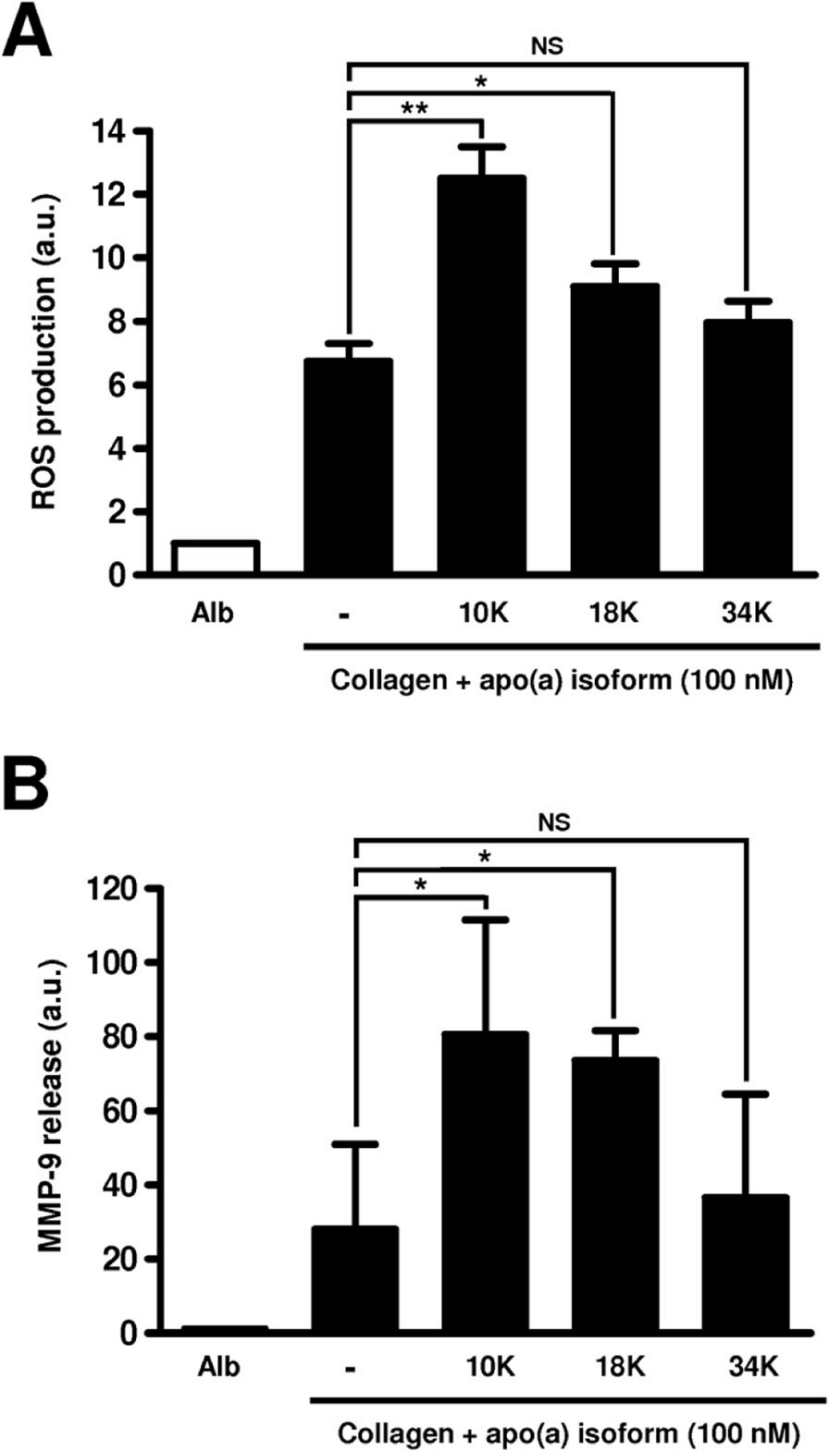
Influence of the number of apo(a) Kringle IV-2 repeats on ROS production and MMP-9 secretion by collagen-primed monocytes. Monocytes were incubated on albumin (Alb) or type I collagen (closed bars) at 37 °C with or without 100 nM 10 K, 18 K, or 34 K apo(a). **a** ROS production evaluated by NBT reduction test. **b** MMP-9 secretion evaluated by ELISA. Results (means ± standard deviations) are expressed as ratios to values obtained on albumin-coated plates. a.u.: arbitrary units, NS: non-significant, *: *p* < 0.05, **: *p* < 0.01

The effects of apo(a) isoforms of different sizes (10 K, 18 K, and 34 K) on monocytes seeded on type I collagen were compared. None of the apo(a) isoforms altered monocyte adhesion to type I collagen (data not shown). ROS release by monocytes after a 2 h-incubation on collagen was significantly increased by both the 10 K and the 18 K isoforms (+ 86%, *p* < 0.01 and + 35%, *p* < 0.05, respectively), the highest stimulating effect being triggered by the 10 K isoform. In contrast, no significant effect was observed in the presence of the 34 K apo(a) isoform (Fig. 3a).

MMP-9 secretion in culture media by monocytes was three times higher (*p* < 0.05) in the presence of 10 K and 18 K apo(a) isoforms than in control conditions (i.e. without apo(a)), whereas it was only 1.3 fold enhanced (non-significant) in the presence of the 34 K apo(a) isoform (Fig. 3b).

### Apo(a)-mediated stimulation of ROS and MMP-9 production by collagen-primed monocytes involves a lysine-dependent mechanism

**Fig. 4:**
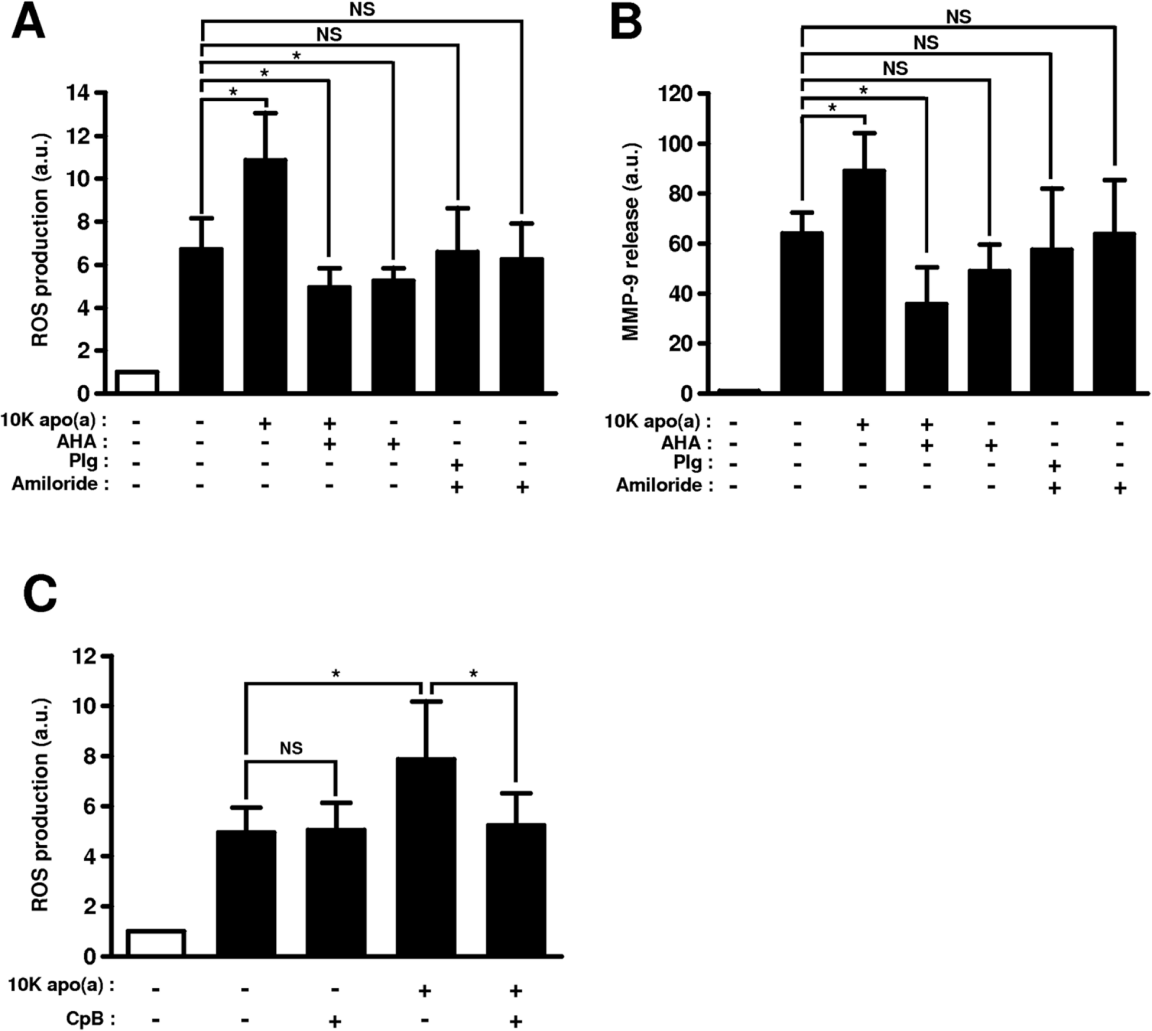
Molecular interactions involved in the apo(a)-mediated activation of collagen-primed monocytes. **a**, **b** Monocytes were incubated at 37 °C on albumin (open bar), or type I collagen (closed bars) with 100 nM 10 K apo(a) and/or 100 mM AHA, or 100 nM plasminogen (Plg) and/or 10 μM amiloride. **c** Monocytes were first preincubated for 1 h at 37 °C with 200 μg/ml carboxypeptidase B (CpB) before being incubated with or without 100 nM 10 K apo(a). Results (means ± standard deviations) are expressed as ratios to the ROS production (**a**) or MMP-9 secretion (**b**) on albumin-coated plates. a.u.: arbitrary units, NS: non-significant, *: *p* < 0.05, **: *p* < 0.01

In order to identify the molecular mechanisms involved in the interaction of apo(a) with monocytes stimulated by type I collagen, experiments were performed using the kringle IV type 2-free apo(a), i.e. 10 K apo(a). First, monocytes seeded on type I collagen were incubated with 100 nM 10 K apo(a) and 100 mM AHA a lysine structural analog used as a competitor for apo(a) lysine binding sites (LBS). The stimulating effect of 10 K apo(a) isoform on ROS and MMP-9 production was completely abolished by AHA, suggesting an LBS-mediated mechanism (Fig. 4a and b). AHA only slightly inhibited collagen-induced monocyte activation in the absence of apo(a) (− 22% ROS production, *p* < 0.05, − 24% MMP-9 production, non-significant).

Second, monocytes were incubated with plasminogen instead of apo(a), in order to evaluate the specificity of apo(a) effects. Plasminogen was considered the most relevant control protein because of the structural homologies between the two molecules. In order to test the LBS-binding effect in functional isolation, the formation of plasmin was prevented with the use of amiloride, an uPA inhibitor. In these conditions, plasminogen had no significant effect on monocyte activation, as evaluated by ROS and MMP-9 production (Fig. 4a and b).

Third, the potential involvement of monocyte membrane proteins susceptible to interact with apo(a) via carboxy-terminal lysine residues was checked by preincubating cells with CpB. Pretreatment of cells with CpB completely abolished the stimulating effect of apo(a) on collagen-primed monocytes (Fig. 4c). 

## Discussion

Apo(a) is a potential good candidate to interact with inflammatory cells (e.g. monocytes) and ECM molecules (e.g. fibronectin and collagens) in the arterial wall, because it is a potent chemoattractant for monocytes, which specifically interacts with α_M_β_2_ integrin [2]. Monocyte activation plays a pivotal role in atheroma plaque formation by releasing ROS and ECM remodelling enzymes like MMP-9. ROS contribute to LDL oxidation and subsequent formation of foam cells, and MMP-9 is involved in ECM degradation, thus contributing to the rupture of atherosclerotic plaques. Our group has previously shown that monocytes were activated by type I collagen through α_x_β_2_ integrin [18]. This study aimed to determine the potential interactions of apo(a) isoforms with collagen-primed monocytes focussing on the role of kringle IV-2 repeats.

Our results show that ROS and MMP-9 production by monocytes stimulated by type I collagen is significantly enhanced by apo(a). This effect is specific of apo(a) since no effect was observed in the presence of plasminogen, a structural analogue of apo(a). Interestingly, the extent of stimulation was inversely proportional to the number of kringle IV-2 repeats, which is consistent with the deleterious role of small size isoforms in atherosclerosis progression described in the literature [10–12]. Furthermore, these data suggest that the increasing number of kringle IV-2 repeats protects against the deleterious effect of apo(a).

According to these results, we could expect that small and high size apo(a) isoforms supported different molecular mechanisms of interaction. Based on apo(a) sequence homology with plasminogen, which interacts with cell receptors via the LBS substructures of its kringle domains [17], we investigated the involvement of apo(a) LBS in this interaction. Functional apo(a) LBS are present in kringle IV-6 to IV-8 and kringle IV-10 domains whereas the LBS substructures of apo(a) kringle IV-2 domains are not functional due to point mutations or deletions [21]. We show here that both the lysine analogue AHA, which blocks the LBS, and CpB, which cleaves carboxy-terminal lysine residues of membrane proteins, abolished apo(a)-induced ROS and MMP-9 production, thus unveiling a lysine-dependent mechanism. A similar interaction has been reported for the adhesion of monocytes to immobilized apo(a) via the α_M_β_2_ integrin [2]. The interaction of apo(a) with monocytes does not rule out the possibility of a direct interaction of apo(a) with collagen in this model, since it has been already proposed that the differential affinity of LBS could mediate the binding of apo(a)/Lp(a) to ECM components such as fibronectin or laminin [22]. However, a moderate inhibition of collagen-induced monocyte activation by AHA was observed in the absence of apo(a), suggesting a direct involvement of collagen lysine residues with monocytes. We have previously described such an interaction between type I collagen and human polymorphonuclear neutrophils [19].

Kringle IV type 2-dependent high size apo(a) isoforms proved unable to further activate monocytes stimulated by collagen, which suggests a molecular size component effect. Becker and al. found in 2003 that the efficiency of Lp(a) assembly in vivo can be modulated by an accessory protein(s) that alters the conformational status of apo(a) [23]. We propose that the increasing number of kringle IV-2 introduces conformational changes that hinders the availability of LBS and prevents specific interactions between apo(a) and monocytes. The observed effect was specific to apo(a) as plasminogen was unable to produce a similar enhancement on collagen-primed monocytes. Note that plasminogen interacts primarily with lysine residues via kringle 1 LBS that is not represented in apo(a) whereas the primary interaction with carboxy-terminal lysine residues involves kringle IV-10 LBS [24]. Further to this subtle difference, apo(a) may also have secondary interactions between its other kringle IV types and monocyte membrane glycoproteins [22]. These integrin-mediated interactions may contribute to the effects observed on monocyte activation. Romagnuolo and al. demonstrated that apo(a) is capable of inhibiting pericellular plasminogen activation. They suggested that the number of kringle IV type 2 repeats does not dictate the level of inhibition [25]. Another study has highlighted that apo(a) isoforms may display polymorphism-linked functional heterogeneity with regard to cell binding and showed that larger Lp(a) isoforms were found to bind with less affinity to THP-1 monocytes [26].

Moreover, these results also suggest that the collagen-dependent enhancement of monocyte proteolytic and inflammatory functions by apo(a) is probably related to a specific effect of type I collagen enabling signalling events that mediate differentiation of peripheral monocytes into resident macrophages [27]. Apo(a) may thus contribute to the continuous state of macrophage activation.

In this study we use r-apo(a), it should be interesting to compare the Lp(a) effect from donors (with different isoform sizes), to see if the same trends hold.

## Conclusions

Our results point to a protective role of kringle IV type 2 copies on the deleterious effect of apo(a) and reinforce the hypothesis of a role for small size apo(a) isoforms in atherothrombosis, through destabilization of atheroma plaque due to ROS and MMP-9 release by monocytes according to a lysine-dependent mechanism. These findings provide a novel insight into a better understanding of apoliproprotein (a) isoforms role in thrombo embolic diseases.

## Abbreviations

AHA: 6-aminohexanoic acid
apo(a): apolipoprotein(a)
CpB: carboxypeptidase B
ECM: extracellular matrix
K: Kringle
LBS: lysine binding site
LDL: low-density lipoprotein
Lp(a): lipoprotein(a)
MMP-9: matrix metalloproteinase-9
ROS: reactive oxygen species
